# The effect of coping with stress in predicting the needs, death anxiety, and depression of patients’ relatives in intensive care units

**DOI:** 10.1186/s12889-026-26407-3

**Published:** 2026-01-28

**Authors:** Havva Kaçan, Şevval Yeyit Bozdemir

**Affiliations:** Department of Mental Health and Psychiatric Nursing, Department of Nursing, Faculty of Health Sciences, Kastamonu University, Kastamonu, Turkey

**Keywords:** Intensive care units, Family, Anxiety, Death, Depression

## Abstract

**Background:**

The study aimed to examine the effects of coping with stress in predicting the needs, death anxiety, and depression of relatives of patients hospitalized in Intensive Care Units.

**Materials and methods:**

A sample of 366 relatives of patients hospitalized in intensive care units was formed, and data were collected face-to-face using a General Information Form, the Needs Scale for Patient Relatives in Intensive Care Units, Turkish Death Anxiety Scale, the Beck Depression Inventory and the Ways of Coping with Stress Scale. The sample consisted of relatives of patients hospitalized in intensive care between September 2021 and June 2022. The data obtained in the study were analyzed using the SPSS (Statistical Package for Social Sciences) for Windows 22.0 program.The data were analyzed using numbers, percentages, means, standard deviations, Pearson Correlation, and regression analysis.

**Results:**

The mean total needs score of the patient relatives was found to be 123.462 ± 24.918, the mean death anxiety score was 42.653 ± 19.881, and the mean depression score was 10.500 ± 8.270. In the dimension of coping styles, the means were as follows: self-confident approach 18.954 ± 4.389, desperate approach 20.820 ± 4.306, submissive approach 15.863 ± 3.162, optimistic approach 15.093 ± 2.788, and seeking social support 9.396 ± 2.251. A positive and statistically significant correlation was found between death anxiety and the total needs score (*r* = 0.393, *p* < 0.01). This finding indicates that as death anxiety increases, the needs level of the patient’s relatives also increases. Conversely, a negative and statistically significant correlation was found between a confident coping approach and the total needs score (*r* = − 0.326, *p* < 0.01). This result shows that as confident coping skills increase, the perceived needs level decreases. Similarly, a negative, strong, and statistically significant correlation was found between an optimistic approach and the total needs score (*r* = − 0.359, *p* < 0.01). This finding reveals that individuals who use optimistic coping strategies more frequently have lower needs levels.

**Conclusion:**

Patients’ families have several needs. Increasing the level of needs of patients’ families leads to increased death anxiety, while decreasing self-confidence and optimistic coping strategies. This demonstrates that a holistic approach, focusing not only on the patients but also on the needs of their families, is crucial for preventing potential psychological symptoms.

## Introduction

Intensive Care Units (ICUs) are healthcare areas where critically ill patients receive advanced medical care and high-technology and multidisciplinary approaches are used [1]. Naturally, all attention is focused on the patient and the disease in intensive care units. However, in this process, the feelings of fear, anxiety, and curiosity of the family waiting outside the unit are ignored; the family is mostly seen only as individuals who provide materials and handle paperwork [1–3]. Family members witnessing the struggle for life of their patients may experience intense stress accompanied by uncertainty, fear, and anxiety, physical and mental fatigue, death anxiety, and depressive symptoms [1, 3–6].

The emotional burden of patients’ families shapes their expectations, needs, and coping mechanisms regarding healthcare services. In this process, information, emotional support, spiritual needs, and basic physical needs are of paramount importance to patients’ families [3]. However, the needs of family members must be taken into account in the treatment and care services provided by health personnel. It is observed that little or no space is given to the subject [7]. The limitations of the intensive care settings, uncertainty about the patient’s condition [8–10], and a focus on the patient and the disease rather than the patient’s relatives [8], can make it difficult to meet these needs. Also, the fear of losing a loved one for the patient’s relatives [10], the uncertainty of the situation, economic problems, role changes, inadequate needs of the patient’s relatives, inability to obtain sufficient information about the patient’s health status, inability to participate in the patient’s care, complex tools and equipment in the intensive care unit, and inability to see the patient adequately are sources of stress and cause anxiety for the patient’s relatives [3]. The fear of losing the patient at any moment and thoughts of grief can cause death anxiety in the patient’s relatives [10]. Relatives of patients in intensive care are negatively affected both psychologically and physically due to the anxiety they experience [4]. The stress that arises during this process hinders their coping skills and increases the risk of encountering psychological problems such as anxiety and depression [11].For this reason, the psychological support provided both makes it easier for them to cope with stress and contributes to the home care processes of patients. Also, relatives of patients whose needs are not adequately met tend to exhibit more desperate and submissive approaches, while those whose needs are met tend to act self-confidently and seek social support [9]. In the study conducted by it was reported that as the level of death anxiety of patient relatives increases, their spiritual well-being levels also increase, which is explained by the fact that individuals who fear losing their patients treated in intensive care tend to turn more to spiritual feelings to cope with increased death anxiety [10]. For this reason, it is stated that the families of patients in intensive care units need support to reduce their death anxiety, provide emotional relief, be informed, be with their patients, offer them support, share their feelings, and meet their individual needs [12].

In our country, studies conducted on providing the necessary information and support to the families of patients in intensive care are quite inadequate [3, 10]. The study aimed to examine the impact of stress management on the prediction of family needs, death anxiety, and depression. To maintain ideal healthcare, patients and their families must be considered as a whole. Nurses must not only address the needs of patients but also support the needs of their relatives within a holistic approach, remembering that the patient and their family are integrated, as stated in nursing philosophy [13]. Recommendations that patients’ relatives receive regular support and counseling within the scope of consultation-liaison nursing, a field of psychiatric nursing, regarding their identified needs and death anxiety, and also to inform the nursing literature with the research results.

For the relatives of patients to navigate this process in a healthy way, intensive care nurses need to focus on two main goals: considering the needs of the relatives in care planning with a holistic care approach [3], and effectively managing stress by meeting these needs. Identifying the needs of the relatives can contribute to preventing psychological symptoms such as death anxiety and depression [11]. Relatives of patients in intensive care units are exposed to intense stressors such as uncertainty, sudden clinical changes, death threats, and loss of control. It is known that during this process, the needs of relatives, death anxiety, and depressive symptoms can increase; this can negatively affect both individual mental health and the patient care process. However, a review of the literature reveals a limited number of studies that address the needs, death anxiety, and depression levels of relatives together and examine how these variables are shaped by their coping styles. Especially, identifying the protective or risk-increasing role of coping strategies on the psychological responses of relatives in the intensive care process is important for both planning psychosocial support interventions and structuring care services with a holistic approach. In this context, this study, which examines the effects of coping styles on needs, death anxiety, and depression in relatives of intensive care patients, is expected to fill a gap in the existing literature and contribute to clinical practices and future intervention-based research.

In the present study, the aim was to examine the effects of coping with stress in predicting the needs, death anxiety, and depression of relatives of patients in intensive care. To this end, the following hypotheses were formulated.

- H0: According to the needs of patient relatives, methods of coping with stress have no effect in predicting death anxiety and depression.
- H1: According to the needs of the patient’s relatives, the methods of coping with stress affect the prediction of death anxiety and depression.

## Methods

### Design

The study had a cross-sectional, correlational, and descriptive design.

### Population and sample

The study was conducted with relatives of patients hospitalized in adult intensive care units at Kastamonu Training and Research Hospital. The sample consisted of relatives of patients hospitalized in intensive care between September 2021 and June 2022. The population consisted of individuals who had at least one family member who cared for patients admitted to the intensive care unit during the last year between the dates of the study. The sample size was calculated using the formula for finite populations [n = N·t²·p·q / d²·(*N* − 1) + t²·p·q] in order to determine a sufficient number of participants to represent the population. In this formula, *n* represents the sample size, N the population size, t the table value corresponding to the selected confidence level, p the probability of occurrence of the characteristic under investigation in the population, q (1 − p), and *d* the accepted sampling error.(Cochran, W. G. (1977). *Sampling techniques* (3rd ed.). New York, NY: John Wiley & Sons).

Based on the data, the number of patients hospitalized in all intensive care units in the last year was 3496. The sample size was calculated as 357. Face-to-face interviews were conducted with 366 relatives of patients between the specified dates. Data collection forms were read and marked by the researchers.

### Data collection

The data collection tools consisted General Information Form, Needs Scale for Patient Relatives in Intensive Care Units, Turkish Death Anxiety Scale, Beck Depression Inventory, and Ways of Coping with Stress Scale.

### General information form

The General Information Form used in the study was prepared by the researchers and consisted of 15 questions on socio-demographic characteristics [3, 10–12]. It is specific to this study and has not been used elsewhere.

### Needs scale for patient relatives in intensive care units (NCPRICU)

The Critical Care Family Needs Inventory (CCFNI) was developed by Molter in 1979 to determine the needs of family members of patients in intensive care units. The scale was developed to emphasize the importance of the needs of families of patients in intensive care and to measure family needs. NCPRICU’s validity and reliability studies have been conducted in many languages and cultures. The validity and reliability study was conducted in Turkish society by Büyükçoban et al. [13]. The Cronbach’s Alpha Value of the scale was determined to be 0.93 in the original study and was 0.92 in the present study [13]. The NCPRICU consisted of 40 statements rated from 1 to 4 (1 = Not important, 2 = Less important, 3 = Important, 4 = Very important). The scale has five subscales: Trust, Information, Closeness, Support, and Comfort. The maximum possible score on the Likert-type scale is 160, and the minimum score is 40. The subscales can also be scored separately. Low total and subscale scores indicate a decrease in needs, while increasing mean scores indicate an increase in needs.

### Turkish death anxiety scale (TDAS)

The Turkish Death Anxiety Scale, developed by Sarıkaya and Baloğlu, consists of 20 items rated on a 5-point Likert scale (0–4), with total scores ranging from 0 to 80; higher scores indicate greater death anxiety [14].

The scale has no reverse-scored items and comprises three subdimensions: uncertainty of death, suffering, and exposure. Factor analyses supported a three-factor structure explaining 67.27% of the total variance, and criterion validity was demonstrated through significant correlations with death anxiety, trait and state anxiety, and depression measures (*p* < 0.01).

### Beck depression inventory (BDI)

The Beck Depression Inventory (BDI), developed by Beck [15] and revised in 1978, is a 21-item self-report scale designed to assess the severity of depressive symptoms. Each item is rated on a 4-point Likert scale (0–3), yielding a total score ranging from 0 to 63, with higher scores indicating greater depression severity [15]. The items cover emotional, cognitive, behavioral, and somatic symptoms of depression, and respondents select the statement that best reflects their feelings during the past week, including the current day. The individual is asked to select and mark one of the four items in each group that best describes how they have felt during the past week, including the current day. The number next to each item (between 0 and 3) indicates the score to be given for that item. A depression score is obtained by summing these scores. The Turkish validity and reliability study of the Beck Depression Inventory was conducted by Hisli [16].

### Ways of coping with stress scale (WCSC)

The Ways of Coping with Stress Scale (WCSC), originally developed by Folkman and Lazarus, is a 4-point Likert-type scale [17]. The shortened Turkish version consists of five subscales: self-confident, desperate, submissive, optimistic, and seeking social support. Higher scores indicate greater use of the corresponding coping style. Items 1 and 9 in the social support subscale are reverse-scored. The scale was first used in Turkey by Siva [18]. The scale demonstrates acceptable reliability, with reported Cronbach’s alpha values ranging from 0.45 to 0.73 across subscales. In the present study, the overall Cronbach’s alpha was 0.82; subscale alpha values were 0.77 (self-confident), 0.74 (desperate), 0.65 (submissive), 0.75 (optimistic), and 0.59 (seeking social support) [19]. In this study, Cronbach’s alpha value for the Ways of Coping with Stress Scale was detected to be 0.82. When examined in terms of factors, Cronbach’s alpha value was detected to be 0.77 for the self-confident approach, 0.74 for the desperate approach, 0.65 for the submissive approach, 0.75 for the optimistic approach, and 0.59 for seeking social support.

### Ethical dimensions of the study

It was approved by the Kastamonu University Training and Research Hospital Clinical Research Ethics Committee (September 22, 2021, 2020-KAEK-143-112).Written permission from the hospital chief physician, and institutional permission from the Kastamonu Provincial Health Directorate. Verbal and written information was provided to the participating first-degree relatives (mother, father, spouse, child), and their written permissions were obtained using the Informed Consent Form. The study was conducted in line with the Helsinki Declaration and informed consent was obtained from the participants.

### Statistical analysis of data

The data obtained in the study were analyzed using the SPSS (Statistical Package for Social Sciences) for Windows 22.0 program. Descriptive statistical methods used in the evaluation of the data were number, percentage, mean, and standard deviation. Kurtosis (Kurtosis) and Skewness (Skewness) values were examined to determine whether the research variables showed a normal distribution. For total need, kurtosis is 0.548, and skewness is -1.027. The kurtosis values of the support, information, closeness, trust, and comfort sub-dimensions are 1.208, 2.135, 0.854, 1.415, and 0.841, respectively, and skewness values are − 0.962, -1.194, -1.056, -1.369, and − 1.036. The kurtosis values for total death anxiety and its sub-dimensions, uncertainty of death, thinking about death, witnessing death and suffering, are − 0.563; -0.565; -0.854; -0.699; and skewness values are − 0.343; -0.48; 0.257 and − 0.63. Depression total kurtosis is 0.745, and skewness is 1.319. For the sub-dimensions of coping with stress, confident approach, desperate approach, submissive approach, optimistic approach, and seeking social support, kurtosis values are 0.425, -0.448, 0.143, -0.359, -0.482, and skewness values are − 0.454, -0.104, -0.268, -0.035, and 0.046. In the relevant literature, a normal distribution is considered when the skewness and kurtosis values of the variables are between + 1.5 and − 1.5 [20] and + 2.0 and − 2.0 [21] It was determined that the variables exhibited a normal distribution. Pearson correlation and regression analysis were applied to the continuous variables of the study.

### Inclusion criteria

Inclusion criteria were having first-degree relative (mother, father, sibling, spouse, child), or having second-degree relative (aunt, uncle, paternal uncle) for patients without first-degree relatives, family members over the age of 18 and under the age of 65, family members with good hearing and comprehension skills, at least literacy, and Turkish as a native language. Family members were selected if the patient’s first 24 h of intensive care unit admission had been completed, and those who agreed to participate in the study voluntarily were included. Furthermore, family members of patients who had been under intensive care monitoring for less than 24 h or more than six months (the needs and expectations of family members who have been in the intensive care unit for more than six months vary as they adapt to the situation), and family members whose mental state (intense anxiety, stress, crying, etc.) made them unsuitable for communication were excluded from the study.

## Results

Table 1 shows the distribution of employees according to their descriptive characteristics. Among the patient relatives who participated in the study, 64.2% were female, 56.0% were primary school graduates, 78.1% were married, and 61.5% were unemployed. In terms of degree of relationship, 62.0% were first-degree relatives and 38.0% were second-degree relatives. Regarding losing a relative, 66.4% responded “yes” and 33.6% responded “no.” A total of 34.4% of patient relatives reported having serious health problems, and 65.6% reported not having serious health problems. The proportion of patient relatives who received information during the intensive care process was 73.8%, while the proportion of those who did not receive information was 26.2%. 60.0% reported receiving information from a physician, and 40.0% from a nurse. Among the individuals responsible for care, 15.0% were mothers, 7.4% were fathers, 4.1% were grandmothers, 7.7% were spouses, 5.2% were children, and 60.7% indicated this as “none.” The average age of the participants was detected to be 45.780 ± 15.401 (min = 18, max = 88).

**Table 1:** Distribution of relatives according to descriptive characteristics

Groups	Frequency (*n*)	Percentage (%)
Sex		
Female	235	64.2
Male	131	35.8
Educational Status		
Primary school	205	56.0
High school	101	27.6
College	60	16.4
Marital status		
Married	286	78.1
Single	80	21.9
Job		
Working	141	38.5
Not working	225	61.5
Degree of Closeness		
1st degree	227	62.0
2nd degree	139	38.0
Relative of a Patient Previously Hospitalized in ICU		
Yes	178	48.6
No	188	51.4
Death-Risk		
Yes	62	16.9
No	304	83.1
Loss of a loved one		
Yes	243	66.4
No	123	33.6
Serious Health Problem		
Yes	126	34.4
No	240	65.6
Duration of Stay in YB		
2–5 Days	181	49.5
6–9 Days	87	23.8
10–13 Days	77	21.0
13 and Above	21	5.7
Getting Information During the ICU Process		
Yes	270	73.8
No	96	26.2
Source of information		
Physician	162	60.0
Nurse	108	40.0
Type of ICU Where the Patient’s Relative was Admitted		
1st degree	114	31.1
2nd degree	128	35.0
General Intensive Care	105	28.7
Cardiovascular Surgery	19	5.2
The Status of Being a Dependent Individual		
Mother	55	15.0
Father	27	7.4
Grandmother/Maternal Grandmother	15	4.1
None	222	60.7
Spouse	28	7.7
Children	19	5.2
	Mean	SD
Age	45.780	15.401

Table 2 shows the descriptive statistics regarding the total mean scores of the scales used in the study. When the mean scores for needs, death anxiety, depression, and styles of coping with stress were examined, the participants’ mean total needs score was detected to be 123.462 ± 24.918 (min = 40.00; max = 160.00). The mean total death anxiety score was 42.653 ± 19.881, and the means according to the sub-dimensions were: uncertainty of death 22.762 ± 10.507, thinking of death witnessing 12.434 ± 7.645, and suffering 7.456 ± 3.550. The total depression score was detected to be 10.500 ± 8.270 (min = 0.00; max = 46.00). In terms of styles of coping with stress, the means were as follows: self-confident approach 18.954 ± 4.389, desperate approach 20.820 ± 4.306, submissive approach 15.863 ± 3.162, optimistic approach 15.093 ± 2.788, and seeking social support 9.396 ± 2.251.

**Table 2:** Mean scores of needs. death, anxiety, depression, and styles of coping with stress

	*N*	Mean	SD	Min.	Max.
Needs Scale Total Score	366	123.462	24.918	40.000	160.000
Support	366	36.533	8.356	13.000	52.000
Information	366	26.511	5.793	9.000	36.000
Relationship	366	23.186	4.993	7.000	28.000
Trust	366	16.355	3.859	5.000	20.000
Comfort​	366	20.877	4.578	6.000	24.000
Death Anxiety Total Score	366	42.653	19.881	0.000	80.000
Uncertainty of Death	366	22.762	10.507	0.000	40.000
Thinking of Death Witnessing	366	12.434	7.645	0.000	28.000
Suffering	366	7.456	3.550	0.000	12.000
Depression Scale Total Score	366	10.500	8.270	0.000	46.000
Self-Confident Approach	366	18.954	4.389	7.000	28.000
Desperate Approach	366	20.820	4.306	11.000	32.000
Submissive Approach	366	15.863	3.162	7.000	24.000
Optimistic Approach	366	15.093	2.788	8.000	20.000
Seeking Social Support	366	9.396	2.251	5.000	14.000

Table 3 shows the correlation analysis between the scores of Needs, Death Anxiety, Depression and Coping Styles. A significant positive correlation was detected between the total death anxiety score and the total needs score (*r* = 0.393, *p* < 0.01). Similarly, significant positive correlations were detected between the sub-dimensions of uncertainty of death (*r* = 0.374, *p* < 0.01), thinking about and witnessing death (*r* = 0.359, *p* < 0.01), and suffering (*r* = 0.320, *p* < 0.01) and the total needs score.

Although there was no significant relationship between depression and the total needs score (*r*=-0.092, *p* > 0.05), only weak but significant negative relationships were observed with the closeness (*r*=-0.123, *p* < 0.05) and trust (*r*=-0.115, *p* < 0.05) sub-dimensions.

Significant negative correlations were detected between the self-confident approach and the total needs score (*r*=-0.326, *p* < 0.01) and all sub-dimensions of coping styles. Similarly, strong and significant negative correlations were found between the optimistic approach and the total needs score (*r*=-0.359, *p* < 0.01) and all sub-dimensions. However, no significant relationship was found between the helpless approach and the submissive approach and the total needs score, and only a weakly significant relationship was found with the support sub-dimension (*r*=-0.109, *p* < 0.05 for the helpless approach; *r* = 0.105, *p* < 0.05 for the submissive approach). No significant relationship was detected between seeking social support and needs scores (*p* > 0.05).

**Table 3:** Correlation analysis between need, death, anxiety, depression, and stress coping styles scores

		Requirement Total	Support	Information	Relationship	Trust	Comfort
Death Anxiety Total	r	0.393**	0.451**	0.407**	0.273**	0.379**	0.186**
	p	0.000	0.000	0.000	0.000	0.000	0.000
The Uncertainty of Death	r	0.374**	0.434**	0.367**	0.263**	0.368**	0.185**
	p	0.000	0.000	0.000	0.000	0.000	0.000
Thinking of Death Witnessing	r	0.359**	0.416**	0.391**	0.239**	0.330**	0.163**
	p	0.000	0.000	0.000	0.000	0.000	0.002
Suffering	r	0.320**	0.344**	0.349**	0.235**	0.325**	0.141**
	p	0.000	0.000	0.000	0.000	0.000	0.007
Depression Total	r	-0.092	-0.030	-0.097	-0.123*	-0.115*	-0.092
	p	0.079	0.566	0.063	0.018	0.028	0.080
Self-Confident Approach	r	-0.326**	-0.352**	-0.298**	-0.283**	-0.285**	-0.210**
	p	0.000	0.000	0.000	0.000	0.000	0.000
Desperate Approach	r	-0.051	-0.109*	-0.042	0.018	-0.012	-0.033
	p	0.334	0.038	0.420	0.731	0.812	0.531
Submissive Approach	r	0.037	0.105*	0.070	-0.017	0.034	-0.087
	p	0.476	0.045	0.180	0.744	0.513	0.096
Optimistic Approach	r	-0.359**	-0.340**	-0.379**	-0.304**	-0.311**	-0.259**
	p	0.000	0.000	0.000	0.000	0.000	0.000
Seeking Social Support	r	0.045	0.040	-0.022	0.081	0.038	0.078
	p	0.394	0.449	0.674	0.121	0.469	0.136

**<0.01; Pearson Correlation Analysis *p<0.05

Table 4 shows the variables affecting the needs of patient relatives. In the regression analysis, the total need score was detected to have a significant and positive effect on death anxiety (β = 0.393; *p* < 0.001). The explanatory power of the model was 15.2% (R² = 0.152), and the model was generally significant (F = 66.571; *p* < 0.001). The increase in death anxiety increased with the increase in need scores.

Similarly, when the effect of the total need score on the self-confident coping style was examined, a negative and significant relationship was detected between them (β = -0.326; *p* < 0.001). The explanatory power of the model was 10.4% (R² = 0.104), and a significant model was obtained (F = 43.425; *p* < 0.001), which suggests that as the need level increases, individuals’ tendency to use the self-confident coping style decreases.

Also, the effect of the total need score on optimism was detected to be negative and significant (β = -0.359; *p* < 0.001). The model’s explanatory power was 12.6% (R² = 0.126), and the model was statistically significant (F = 53.787; *p* < 0.001), which suggests that individuals with higher need levels tend to use optimistic coping less.

**Table 4:** Variables affecting the needs of patient relatives

Dependent Variable	Unstandardized Coefficients		Standardized Coefficients	t	*p*	95% Confidence Interval	
	B	SE	β			Min	Max
Death Anxiety Total	0.314	0.038	0.393	8,159	**0**,**000**	0.238	0.389
*Dependent Variable = Death Anxiety Total, *R = 0.393; R* ^*2*^ *=0.152; F = 66.571; p = 0.000; Durbin Watson Value = 0.139*							
Self-Confident Approach	-0.058	0.009	-0.326	-6,590	**0**,**000**	-0.075	-0.040
*Dependent Variable = Self-Confident Approach, *R = 0.326; R* ^*2*^ *=0.104; F = 43.425; p = 0.000; Durbin Watson Value = 0.591*							
Optimistic Approach	-0.040	0.005	-0.359	-7,334	**0**,**000**	-0.051	-0.029
*Dependent Variable = Optimistic Approach, *R = 0.359; R*^*2*^*=0.126; F = 53.787; p = 0.000; Durbin Watson Value = 0.275*							

*p<0.05

## Discussion

Significant and positive relationships were detected between the need levels of relatives of patients in Intensive Care Units (ICU) and death anxiety (β = 0.393; *p* < 0.001). The analysis results show that need scores significantly predict death anxiety, and the explanatory level of the model was 15.2% (R² = 0.152). This result shows that death anxiety increases when the needs of relatives of patients are not met or are not met adequately. This can directly affect the psychological states of family members, especially in environments such as ICUs where uncertainty, isolation, and life-threatening situations prevail. This result is parallel to those of many studies in the literature. For example, the study by Görücü reported that the death anxiety of individuals with relatives of patients in intensive care is significantly related to their spiritual well-being [10]. Family members experience more anxiety when thinking about the death of their patients, and their need for spiritual support during this process also increases. Likewise, Chiang et al. emphasized that relatives of patients in the ICU experienced high levels of stress and anxiety, and that this was related to unmet needs such as information, support, and comfort [22]. The same study also reported that a short-term cognitive-behavioral psychoeducation program offered to family members reduced anxiety, stress, and depression levels, indicating that meeting the needs of relatives reduced psychological burden. Another study by Naef et al. revealed that symptoms of depression, anxiety, and post-traumatic stress experienced by family members after ICU were negatively correlated with their satisfaction with the intensive care process [23]. In other words, as the perception that needs were adequately met increased, psychological distress decreased. The results also show that the death anxiety levels of patients’ relatives are generally moderate. The highest mean score in the “uncertainty of death” subscale, in particular, suggests that uncertainty has a strong psychological impact on individuals. The scores in the “thinking about and witnessing death” and “suffering” subscales are also notable, demonstrating that witnessing the death process and related mental images have the potential to create anxiety in individuals. Furthermore, the significantly high mean depression score suggests that the intensive care process triggers not only death anxiety but also depressive symptoms in patients’ relatives. These results highlight the necessity of psychosocial support interventions for patients’ relatives and the importance of informative and supportive approaches, particularly for coping with uncertainty. Kynoch et al. similarly detected that family members’ anxiety and depression levels are significantly linked to unmet needs and inadequate support [24]. Hamid and Mat Nor supported the positive effects of meeting families’ needs on stress, anxiety, and depression in his study [25]. This is consistent with our study results. Studies show that failure to meet basic needs, particularly those related to information, emotional support, and close contact with the patient, can further increase families’ death anxiety.

The regression analysis yielded a negative and significant relationship was detected between the need levels of individuals who are relatives of patients in the intensive care unit (ICU) and their assertive coping style (β = -0.326; *p* < 0.001), consistent with various results in the literature, which suggests that as individuals’ needs increase, they become under psychological pressure and for this reason move away from active and assertive coping strategies. While the model’s explanatory power of 10.4% suggests that need levels have a certain but limited impact on coping styles, it does reveal a significant psychosocial relationship. These results directly align with the study conducted by Özçelik and Erdoğan [26] with relatives of patients in ICUs in Turkey. The study detected that when basic needs such as information, support, and reassurance were not met, relatives used more desperate and submissive coping styles, whereas when these needs were met, they used more self-assured and problem-focused coping styles. This suggests that unmet needs weaken an individual’s reliance on internal resources and lead to passivity [26]. Similarly, in a study conducted in Nigeria by Olabisi et al. [11], they reported that task-focused coping styles were negatively correlated with depression, anxiety, and stress levels. When individuals used task-focused and self-confident coping styles more, a decrease in psychological symptoms was observed. However, it was found that individuals with high needs were more likely to employ emotion-based or avoidance-based methods instead of these coping styles [11]. In a review study by Rückholdt et al. [27], it was shown that avoidant and submissive coping styles were associated with intense psychological stress, whereas active coping methods were positively associated with psychological resilience. This study suggests that factors such as social support, previous ICU experience, and individual awareness also shape coping skills [27]. Also, Butler et al. [28] demonstrated that the coping styles of relatives of ICU patients are closely related to personality traits, level of social support, and psychological history (history of anxiety, depression). This study also detected that individuals with “maladaptive” or “avoidant” coping profiles were less likely to use confident and task-oriented strategies [28].

In the study, the need total score had a negative and significant effect on optimistic coping style (β = -0.359; *p* < 0.001), suggesting that as an individual’s need level increases, their tendency to approach events with an optimistic perspective decreases. The explanatory power was significant at 12.6% (R² = 0.126). This finding suggests that individuals receiving treatment in high-stress environments, such as intensive care, may experience a decline in their hopes for the future and positive coping tendencies if their needs are not adequately met. This result differs from some studies in the literature but is consistent with others. For example, a study by Nadig et al. [29] reported that optimism in family members of patients recovering from mechanical ventilation in intensive care units is a protective factor against psychological distress. However, the important point emphasized here is that high optimism is already associated with low stress levels; in other words, as need levels rise and remain unmet, this protective optimistic effect diminishes [29]. Similarly, The literature has found that coping strategies among relatives of patients in intensive care units are related to psychological processes. For example, one study found that family members mostly used emotionally focused and maladaptive coping strategies, and a decrease in appropriate coping strategies was associated with psychological stress and negative emotions [30]. In their studies to determine the levels of hope and spiritual well-being of relatives of intensive care patients, Kant et al. found that the levels of hope and spiritual well-being of the relatives of patients were high and that spiritual well-being increased the level of hope among the relatives of patients [31].Also, a study by Shao found a significant relationship between illness uncertainty and coping styles among families of sepsis patients in intensive care. This study found a negative correlation between positive coping styles and low uncertainty, suggesting that meeting needs and reducing uncertainty may increase optimistic coping tendencies [32].

### Limitations of the study

This study has some limitations. The large number of scales and the fact that data were collected from relatives of patients waiting in front of intensive care units may have affected the participants’ attention and response levels. In addition, the fact that the study was conducted in a single center with a limited sample, has a cross-sectional design, and data were collected via self-report limits the generalizability and causal interpretation of the findings.

## Conclusion

Based on a holistic perspective, the needs of individuals who are relatives of patients in the intensive care unit (ICU) can be said to play a significant role in their stress coping processes. When these needs are not adequately met, the resulting anxiety and depressive symptoms reduce individuals’ self-esteem, leading to a shift in coping styles to more passive, avoidant, and emotion-based styles. For this reason, healthcare professionals must identify the needs of family members early and develop supportive approaches to address these needs to increase individuals’ psychological resilience and encourage more active coping strategies. Holistic assessments and necessary measures must be conducted to meet the physical, psychological, and psychosocial needs of relatives of patients receiving intensive care. This approach aligns with the family-centered approach and forms a fundamental cornerstone in providing quality healthcare. The results of the present study highlights the importance of addressing not only the treatment and care needs of patients but also the holistic approach of patients, including their families and social environments. In this context, it is recommended to increase similar studies focusing on the needs of patient relatives and to develop institutional or individual interventions for these problems detected in clinical settings.

These findings indicate that the relatives of patients in intensive care are largely middle-aged, female, married, and unemployed; the majority are first-degree relatives; and a significant portion have experienced loss. These characteristics suggest that these factors may be decisive in influencing the information needs, psychological responses, and coping processes of the patients’ relatives. he relationships between all demographic characteristics and the scales could not be examined in detail; however, it is recommended that these relationships be addressed in future studies.

In conclusion, identifying the needs of patient relatives in intensive care units at an early stage and developing supportive approaches to these needs strengthens the psychological resilience of individuals, enables them to cope with stress more healthily, and forms the basis of a quality, family-centered care approach. It is important for nurses and healthcare professionals to support the psychological health of family members with an empathic approach during the intensive care process. Multi-center, qualitative studies are needed in this field, and effective social support systems for family caregivers need to be established in Türkiye.

## Data Availability

The data that supports the findings of this study is available from the corresponding author upon reasonable request.
